# Phenotypic evolution in durum wheat (*Triticum durum* Desf.) based on SNPs, morphological traits, UPOV descriptors and kernel-related traits

**DOI:** 10.3389/fpls.2023.1206560

**Published:** 2023-08-28

**Authors:** Stefania Marzario, Rita Sica, Francesca Taranto, Fabio Fania, Salvatore Esposito, Pasquale De Vita, Tania Gioia, Giuseppina Logozzo

**Affiliations:** ^1^ School of Agricultural, Forestry, Food and Environmental Sciences, University of Basilicata, Potenza, Italy; ^2^ Institute of Biosciences and Bioresources (CNR-IBBR), Bari, Italy; ^3^ Department of Agriculture, Food, Natural Resources, and Engineering (DAFNE) - University of Foggia, Foggia, Italy; ^4^ Council for Agricultural Research and Economics (CREA), Research Centre for Cereal and Industrial Crops (CREA-CI), Foggia, Italy

**Keywords:** *Triticum durum* (Desf.), DAPC, molecular marker, phenotypic traits, PCAmix

## Abstract

Durum wheat is a worldwide staple crop cultivated mainly in the Mediterranean basin. Progress in durum wheat breeding requires the exploitation of genetic variation among the gene pool enclosed in landraces, old cultivars and modern cultivars. The aim of this study was to provide a more comprehensive view of the genetic architecture evolution among 123 durum wheat accessions (41 landraces, 41 old cultivars and 41 modern cultivars), grown in replicated randomized complete block in two areas, Metaponto (Basilicata) and Foggia (Apulia), using the Illumina iSelect 15K wheat SNP array and 33 plant and kernel traits including the International Union for the Protection of new Varieties of Plants (UPOV) descriptors. Through DAPC and Bayesian population structure five groups were identified according to type of material data and reflecting the genetic basis and breeding strategies involved in their development. Phenotypic and genotypic coefficient of variation were low for kernel width (6.43%) and for grain protein content (1.03%). Highly significant differences between environments, genotypes and GEI (Genotype x Environment Interaction) were detected by mixed ANOVAs for agro-morphological-quality traits. Number of kernels per spike (h^2^ = 0.02) and grain protein content (h^2^ = 0.03) were not a heritability character and highly influenced by the environment. Nested ANOVAs revealed highly significant differences between DAPC clusters within environments for all traits except kernel roundness. Ten UPOV traits showed significant diversity for their frequencies in the two environments. By PCAmix multivariate analysis, plant height, heading time, spike length, weight of kernels per spike, thousand kernel weight, and the seed related traits had heavy weight on the differentiation of the groups, while UPOV traits discriminated moderately or to a little extent. The data collected in this study provide useful resources to facilitate management and use of wheat genetic diversity that has been lost due to selection in the last decades.

## 1 Introduction

Wheat represents the main source of food, feed, and industrial raw materials and is grown on about 222 million hectares worldwide, with a world production of around 771 million tons in 2020/2021 sowing season (FAO, 2021). Durum wheat (*Triticum durum* Desf.), an allotetraploid (2n = 4x = 28, AABB), is a worldwide staple crop cultivated mainly in the Mediterranean Basin and is the second most important wheat type after common bread wheat (Nazco et al., 2012; Paux et al., 2012; Martínez-Moreno et al., 2020). After Canada, Italy represents the second biggest producer of durum wheat in the World due, partly, to the economic relevance of the pasta industry. The wheat importance was linked to its wide adaptation to local environments but also to the high yields resulting from intensive breeding (De Santis et al., 2018).

Tetraploid wheat was domesticated about 10000 years ago in the Fertile Crescent. The wild progenitor has been identified as the wild emmer (*Triticum turgidum* ssp. *dicoccoides*), which gave rise to emmer (*T. turgidum* ssp. *diccoccum*), the first domesticated tetraploid wheat (Zohary et al., 2012). About 2000 years later, durum wheat appeared in the Near East and began to replace its ancestor, emmer, becoming the major cultivated form of tetraploid wheat (Zohary et al., 2012). From the Fertile Crescent, durum wheat spread throughout the Mediterranean, East Africa, and Asia (MacKey, 2005). For this reason, the wheat populations grown nowadays are very different from their wild progenitors and from domesticated and cultivated populations. In fact, domesticated populations differ from progenitors by loss of spike shattering, loss of tough glumes, increased seed size, reduced number of tillers, more erect growth, and reduced seed dormancy (Gioia et al., 2015; Maccaferri et al., 2019). Simultaneously with the expansion of the cultivation area, the durum wheat genetic selection began, initially as a consequence of the actions of the farmers who simply choose the best seeds for the following season, and after realized by breeders following the genetic laws with the main aim to obtain populations better adapted to different environments and with higher yield and quality (De Vita et al., 2007; Mefleh et al., 2019; Martínez-Moreno et al., 2020; Royo et al., 2020). During the 20th century, artificial hybridization and dip selection pressure for commercial purposes, grain yields, shorter stature and early maturity, radically changed the characteristics of durum wheat (De Vita et al., 2007; Kabbaj et al., 2017).

At the end of 1960s several varieties selected from mediterraneum typicum (Grignac, 1965; Bozzini et al., 1970) durum wheat landraces dominated the crop (Marzario et al., 2018). Later, with the synergy between breeding and management, the attention was focused on the exploitation of the *Rht* genes that caused the reduction in stature (Giunta et al., 2007; Mefleh et al., 2019). In the following years, the landraces and old cultivars were completely replaced by modern cultivars and consequently agronomic practices changed. Landraces and old cultivars, being more suitable to low-input systems, were able to enhance marginal areas where the potential of modern cultivars was not expressed. Instead, the modern cultivars took advantage of high sowing rates and high external input rates (Dwivedi et al., 2016; Marzario et al., 2018; Giunta et al., 2019; Kyratzis et al., 2019; Mefleh et al., 2019). This has led to a loss of biodiversity, indeed the pool of the Mediterranean landraces is regarded as the laugher source of genetic diversity due to its level of polymorphism (Nazco et al., 2012; Alemu et al., 2020; Royo et al., 2020).

Luckily, before becoming obsolescent, landraces and old cultivars were collected ex situ in regional, national and international genebanks and in the last few years these precious genetic resources were reintroduced in cultivation or in plant breeding programs (Marzario et al., 2018; Kyratzis et al., 2019).

In plant breeding programs, agro-morphological characterization represents the first step towards the utilization of genetic resources. In order to underline the differences due to durum wheat history, several studies characterized durum wheat collections both by morphological traits (Spagnoletti Zeuli and Qualset, 1987; Spagnoletti Zeuli et al., 1988; Spagnoletti Zeuli and Qualset, 1990; De Vita et al., 2007; Aghaee et al., 2010; Zarkti et al., 2012; Patil et al., 2013; Marzario et al., 2018; Gharib et al., 2021; Ouaja et al., 2021; Phogat et al., 2021; Shaygan et al., 2021) and by different type of molecular markers (Maccaferri et al., 2005; Ren et al., 2013; Marzario et al., 2018; Fayaz et al., 2019; Fiore et al., 2019; Maccaferri et al., 2019; Taranto et al., 2020; Shaygan et al., 2021; Condorelli et al., 2022; Taranto et al., 2022). Results of the various molecular diversity studies by single nucleotide polymorphism (SNP) array platforms (Semagn et al., 2021) and durum wheat reference genomes (Maccaferri et al., 2019) shed some light on the impact of plant breeding on wheats’ genetic diversity (Sthapit et al., 2020; Taranto et al., 2020; Taranto et al., 2021; Taranto et al., 2022).

Although variations in morphological and yield related traits, quality traits (kernel size and shape traits as kernel length, kernel width, kernel area, kernel thickness and kernel roundness that influences the thousand kernel weight and the quality of semolina), agro-ecological adaptation, abiotic stresses and resistance to pests are very important parameters in plant breeding programs, the durum wheat gene pool remains poorly characterized to date (Troccoli and Di Fonzo, 1999; Gegas et al., 2010; Desiderio et al., 2019; Fayaz et al., 2019).

There was evidence that the modern cultivars have a uniform large seed size, while the durum wheat landraces have a greater diversity. Larger kernels not only impact on grain yield but also could have favorable effects on seedling vigor and early growth; moreover, the market and industry requirements are for almost spherical grains (Liu et al., 2017; Desiderio et al., 2019).

A lot of agro-morphological traits are quantitative with polygenic variation (Falconer, 1981) and highly influenced by genotype, environment and their interactions as well as by dominance, additive and epistatic interactions (Sharma et al., 2003; Novoselovic et al., 2004; Patil et al., 2013). Thus, for plant breeders it is important to investigate and know them to establish a breeding program (Falconer, 1981). Broad-sense heritability was defined as the ratio of genotypic variance to the phenotypic one and in general, it was low for traits with agronomic importance since these characteristics were influenced by a large number of genes. Moreover, correlations among traits and genotype x environment interactions, made the work more difficult for breeders (Yagdi and Sozen, 2009).

The objectives of this study were to provide a more comprehensive view of the genetic architecture evolution of durum wheat by integrating SNP genotyping, phenotyping and pedigreed varieties in order to: a) detect pattern of diversity of 123 durum wheat accessions labeled as landraces, old and modern cultivars; b) investigate the genotype x environment interactions (GEI) and traits heritability for identifying characters useful for genetic improvement; c) compare the durum wheat genetic resources with pedigreed varieties for knowing how much genetic diversity is still keep and available for breeding works.

## 2 Materials and methods

### 2.1 Plant materials

The analysed durum wheat (*Triticum durum* Desf.) collection included a set of one-hundred-twenty-three accessions, originated in different geographical areas (**Supplementary Material 1**), and made available by Research Centre for Cereal and Industrial Crops (CREA-CI), Foggia, Italy (Taranto et al., 2020). The collection was composed of three groups: 41 Italian landraces (LR), 41 old cultivars (OC) and 41 modern cultivars (MC) obtained by randomly stratified samples by groups from Taranto et al. (2020) collection. The landraces group also included two accessions of *Triticum turgidum* subsp. *turanicum* Jakubz (KAMUT_CREA and KAMUT_MOL_DV). The accessions were grown during the growing season 2018/2019 in South Italy in two areas: Metaponto (A.A.S.D. Pantanello of ALSIA, MT, Basilicata, 40° 23’ 27.7’’ N, 16° 47’ 15.1” E) and Foggia (Apulia, 41° 27’ 17.2” N, 15° 29’ 59.5” E). The data of maximum and minimum temperature and rainfall for the growing season (November to July) were obtained from the nearest weather station located, as shown in **Figure S1** (**Supplementary Material 2**). The soil at the two locations is a fertile coarse lime soil in Metaponto and a clay-loam in Foggia. The field experiments were conducted as a randomized complete block design with three replicates. Ten seeds for each accession in each replicate were sown in a single row plot (1 m long, 0.3 m apart). Triple superphosphate (TSP, 46% P2O5) fertilizer was applied as basal fertilization before sowing. Standard conventional agronomic management were applied to the experimental fields. At maturity ten main spikes with well-developed grains were randomly collected from each accession and replicates. The accessions were manually harvested and shelled to avoid seed contamination.

### 2.2 Molecular characterization

In order to estimate molecular diversity, the collection was characterized by SNPs. As reported in Taranto et al. (2020), DNA was extracted from leaves by applying the CTAB method. Genotyping was performed by Trait Genetics (Gatersleben, DE) using the Illumina RiSelect 15K wheat SNParray, which contains 13,600 highly informative gene-associated SNP markers (Muqaddasi, 2017) and is an optimized and reduced version of the 90K iSELECT SNP-chip described by Wang et al. (2014). SNP quality control was performed using Plink v1.07 (Chang et al., 2015). SNPs with a minor allele frequency (MAF) of<1% and a call rate of >10% were excluded from the analysis.

### 2.3 Agro-morphological characterization

The agro-morphological characterization was performed using 33 morphological traits. 18 out of 33 traits were detected according to descriptors for wheat defined by IBPGR (International Board for Plant Genetic Resources, 1985 and 2009; https://www.bioversityinternational.org/e-library/publications/detail/descriptors-for-wheat-revised/), and were reported in **Table 1**.

**Table 1:** Agro-morphological quantitative traits detected on the collection of 123 *ex situ* durum wheat accessions, abbreviation codes, type of trait (QN=quantitative) and corresponding unit.

Traits	Abbreviation codes	Type	Unit
Heading date (from 1st April)	HD	QN	days
Plant height	PH	QN	cm
Awn length	AwnLen	QN	cm
Spike length	SpkLen	QN	cm
Spikelets number/spike	SpktSPK	QN	number
Number of kernels/spike	KerSPK	QN	number
Weight of kernels/spike	KerWgtSPK	QN	g
1000 kernels weight	TKW	QN	g
Kernel roundness	KerRou	QN	DN
Kernel area	KerAre	QN	mm2
Kernel length	KerLen	QN	mm
Kernel width	KerWid	QN	mm
Kernel thickness	KerTck	QN	mm
Blackstain Blackpoints	BP	QN	%
Yellow berry	YBer	QN	%
Grain Protein Content	GPC	QN	%/ss
Sedimentation SDS test	SDS	QN	%
Total Carotenoid Content	TCC	QN	%/ss

Heading date was observed during the growing season, while plant height was measured at harvesting; all the remaining traits were recorded in post harvesting. Kernel traits such as 1000 kernels weight, kernel roundness, kernel area, kernel length, kernel width, kernel thickness, blackstain blackpoints and yellow berry were acquired by a system composed of a fast-modified flatbed scanner and the related software (SeedCount SC5000 Image Analysis System; Next Instruments, Australia). As suggested in Armstrong et al. (2003), kernel roundness value was calculated using the following dimensionless equation:

Roundness = (Width/Length + Thickness/Length + Thickness/Width)/3

The wheat kernels that had been individually measured were compared to their respective Digital Image Analysis (DIA) values and a small SeedCount Roundness adjustment equation was generated.

Semolina technological traits, namely sedimentation-SDS, grain protein content and total carotenoid content were evaluated on 5 g of 0.5 mm semolina for each sample obtained by a laboratory mill (CT193 Cyclotec ™ - FOSS), using near-infrared spectroscopy Rapid Content ™ - FOSS XDS (NIR, Infratec 1241 Analyzer, Foss, Hillerod, Denmark, ICC159).

The remaining 15 morphological traits were detected according to descriptors for durum wheat defined by (U.P.O.V. (International Union for the Protection of New Varieties of Plants, 2012) - (https://www.upov.int/educes/tgdocs/en/tg120.pdf), as reported in **Table 2**. Flag leaf glaucosity of sheath and flag leaf glaucosity of blade were both scored at the half of the heading, while the other traits were measured in post-harvesting.

**Table 2:** Morphological UPOV traits recorded on the collection of 123 *ex situ* durum wheat accessions, abbreviation codes, type of trait (QN=quantitative, QL=qualitative and PQ=pseudo qualitative), levels of expression and measuring period.

Descriptor	Abbreviation codes	Type	Level of expression	Measuring period
Flag leaf: glaucosity of sheath	FGSGls	QN	(1) absent or very weak; (3) weak; (5) medium; (7) strong; (9) very strong	Pre-Harvesting
Flag leaf: glaucosity of blade (lower side)	FGBGls	QN	(1) absent or very weak; (3) weak; (5) medium; (7) strong; (9) very strong	Pre-Harvesting
Lower glume: shape (spikelet in mid-third of ear)	GluShp	PQ	(3) ovoid; (5) elongate; (7) definitely elongate	Post-Harvesting
Lower glume: shape of shoulder (spikelet in mid-third of ear)	GluSShp	PQ	(1) curved; (3) slightly curved; (5) straight; (7) elevated; (9) elevated with second point present	Post-Harvesting
Lower glume: shoulder width (spikelet in mid-third of ear)	GluSWid	QN	(3) narrow; (5) medium; (7) broad	Post-Harvesting
Lower glume: length of beak (spikelet in mid-third of ear)	GluBLen	QN	(1) very short; (3) short;(5) medium; (7) long; (9) very long	Post-Harvesting
Lower glume: shape of beak (spikelet in mid-third of ear)	GluBShp	QN	(1) straight; (3) slightly curved; (5) moderately curved; (7) strongly curved	Post-Harvesting
Lower glume: pubescence of external surface (spikelet in mid-third of ear)	GluPub	QL	(1) absent; (9) present	Post-Harvesting
Awns: color	AwnCol	PQ	(1) whitish; (2) light brown; (3) brown; (4) black	Post-Harvesting
Spike: color (at maturity)	SpkCol	PQ	(1) white; (2) slightly colored; (3) strongly colored	Post-Harvesting
Spike: shape	SpkShp	QN	(1) tapering; (2) parallel sided; (3) semi-clavate; (4) clavate; (5) fusiform	Post-Harvesting
Spike: density	SpkDns	QN	(3) lax; (5) medium; (7) dense	Post-Harvesting
Kernel: shape	KerShp	QN	(3) ovoid; (5) slightly elongate; (7) elongate	Post-Harvesting
Kernel: length of brush hair in dorsal view	KerBrLen	QN	(3) short; (5) medium; (7) long	Post-Harvesting
Kernel: coloration with phenol	KerPhe	QN	(1) absent or very weak; (3) weak; (5) medium; (7) strong; (9) very strong	Post-Harvesting

The phenol color reaction was performed following the UPOV protocol. One hundred kernels from each accession and for each replication were put in Petri dishes and soaked in tap water for 16 to 20 h. After draining and removing surface water, the kernels were placed with crease downwards and ¾ covered by a 1 percent Phenol-solution (freshly made up). The degree of kernels’ color reaction was evaluated after 4 h at room temperature and in daylight (out of direct sunshine) and classified according to the level of expression shown in **Table 2**.

### 2.4 Statistical analysis

#### 2.4.1 SNP data analyses

The population structure within the collection, based on SNPs data, was examined by Discriminant Analysis of Principal Components (DAPC, Jombart and Collins, 2017) implemented in R v. 4.2.0 (R Core Team, 2022) using *adegenet* package v. 2.1.6 (Jombart, 2008; Jombart, 2015). This multivariate method identifies and describes clusters of genetically related individuals, optimizing variance between groups and minimizing variation within clusters. We used the *find.clusters* function to identify clusters and *kmeans* function, a clustering algorithm which finds a given number (*k*) of groups. *Kmeans* runs sequentially with increasing values of *k*, and different clustering solutions are compared using Bayesian Information Criterion (BIC). The optimal number of clusters was indicated as the value of k above which the BIC value decreased or increased. The *dapc* function describes the relationships between the identified clusters. The optimal number of PCs was determined with *optim.a.score* function. The results obtained were plotted in a scatterplot of the first and second linear discriminants of DAPC.

To estimate the divergence between the clusters identified by DAPC analysis, pairwise FSTs were calculated according to Weir and Cockerham (1984) with *pairwise.WCfst* function of R *hierfstat* package (Goudet and Jombart, 2015). The range of the FST was from 0 to 1, where FST=0 indicated that the subpopulations were identical, instead FST=1 indicated that subpopulations were different.

R package *LEA* was used to estimate individual admixture coefficients (Frichot and François, 2015). Assuming K ancestral populations, the R function *snmf* provides least-squares estimates of ancestry proportions rather than maximum likelihood estimates. This function also includes the entropy that can help to choose the number of ancestral populations that best explains the genotypic data (Frichot et al., 2014). The result of the simulation was plotted with the *barplot* function of R package *ggplot2* (Wichkham, 2016).

#### 2.4.2 Morphological quantitative data analyses

A linear mixed model was employed, using the R package *metan* developed by Olivoto et al. (2019), by considering the genotypic effects as random to calculate the components of the variance useful to obtain the genetic parameters.

In addition, differences of 18 out of 33 quantitative agro-morphological traits were performed by analysis of variance (ANOVA) based on different sources of variation: between environments, between DAPC clusters within the environments and between accessions within DAPC clusters and environments. Since the ANOVA showed significant differences between environments, it was decided to proceed by analyzing the two environments separately. The variability between the two environments and among the three groups of accessions in each environment was evaluated considering both quantitative agro-morphological and UPOV-traits.

Univariate statistics including means, minimum, maximum and coefficient of variation, computed by MEANS procedure of SAS OnDemand for Academics (SAS Institute Ltd., North Carolina, USA), were used to describe the variability of the 18 quantitative agro-morphological traits among the five clusters identified by previous analysis for both the environments.

#### 2.4.3 UPOV data analyses

The frequencies for the 15 UPOV-traits were calculated by SAS FREQ procedure and tested with Pearson chi-squared test (χ2).

The Shannon’s diversity index (Shannon and Weaver, 1949) was estimated to determine the phenotypic diversities across environments considering the 15 UPOV phenotypic traits (1):


(1) $$H'\;=\;-\sum_{i}^{n}p_{i}\;ln\;p_{i}$$

wre *p_i_* is the proportion of traits belonging to the *i*th type of class and *n* is the number of the phenotypic classes for a trait. Since different numbers of phenotypic classes were recognized among the traits, this index was standardized by converting it to the relative index, H’ estimated, by dividing it with *H_max_* = *ln*(*n*) as in formula (2):


(2) $$H^{'}=\frac{-\sum_{i}^{n}p_{i}\;ln\;p_{i}}{H_{max}}$$

which ensured all H’ values to be in the range of 0 – 1. According to this index, diversity level is defined as high (H’ > 0.60), intermediate (0.40< H’< 0.60) or low (0.10< H’<0.40) (Eticha et al., 2005). The index was calculated by Microsoft Excel™ software.

#### 2.4.4 Combined morphological and UPOV data analyses

Multivariate analysis of mixed data (numerical and categorical variables) was carried out to determine the overall morphological traits distinctiveness for all the 33 morphological traits using the PCAmix function of R package *PCAmixdata* (Chavent, 2017b; Chavent et al., 2017a). PCAmix by its four outputs (principal component map, correlation circle, level map and squared loading plot) analysed the pattern of similarities between durum wheat genotypes, the pattern of linear links between the 18 quantitative variables, the pattern of proximities between the levels of the 15 categorical variables and the plot of the variables (numerical and categorical) according to their squared loadings giving the pattern of links between the variables regardless of their type (quantitative or categorical) (Chavent, 2017b; Chavent et al., 2017a).

## 3 Results

### 3.1 Molecular characterization of the collection

**Figure 1:**
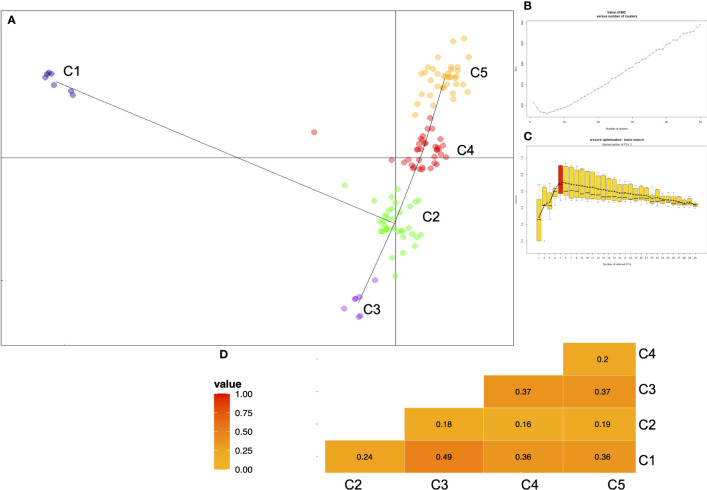
**(A)** Scatterplot of the first two principal components of the DAPC applied on 123 ex situ durum wheat accessions including 41 landraces, 41 old and 41 modern cultivars. Minimum spanning tree connects the five groups. **(B)** Graph of BIC values for increasing values of k and **(C)** plot of the a-score which is simply the difference between the proportion of successful reassignment of the analysis (observed discrimination) and values obtained using random groups (random discrimination) appropriate number of PCs (principal components) retained without overfitting the data. **(D)** Differentiation indices (FST) between all pairwise combinations of clusters identified by DAPC analysis.

The population structure within the durum wheat collection was examined through DAPC analysis based on 1907 SNP data to describe how the accessions were related to each other. The optimal number of clusters individuated by BIC analysis was five genetic clusters. The scatter plot of the two principal components of DAPC is presented in **Figure 1** and the distribution of the accessions in each cluster according to the type was reported in **Table 3** and **Supplementary Material 1**. The first cluster (C1) was composed of all Timilia (n=6; five LR and one OC) and Marzellina Saccone accessions. This cluster was related only with the second cluster (C2), composed of 38 accessions (n=8 OC and n=30 LR). C2 consisted of 71% of the Sicilian landraces such as all the accessions of Russello (n=6) and Scorsonera (n=2). It also included 80% of the Sardinian landraces and eight accessions labeled OC: two, Aziziah and Kyperounda, originated from selected *syryacum* typicum (lower height, higher earliness, higher tillering and shorter awns than *mediterraneum* typicum) lines of Near east and Morocco, four obtained from *Triticum aestivum* crosses and Vera obtained from Eiti-6 x Russello (**Supplementary Material 1**). Two Kamut (*Triticum turgidum* subsp. *turanicum* Jakubz) landraces clustered in this group too, near to the North Africa landraces Jean Rhetifah and Jenah Kottifa.

**Table 3:** Number of accessions per type in the five clusters obtained by DAPC based on 1907 SNPs for 123 *ex situ* durum wheat accessions.

Type	Cluster					Total
	C1	C2	C3	C4	C5	
Landraces (L)	6	30		5		41
Old cultivars (OC)	1	8	7	24	1	41
Modern cultivars (MC)				7	34	41
Total	7	38	7	36	35	123

C2 was related also with C3 and C4. C3 was composed of all the accessions of Dauno (n=5 of Dauno III and n=2 of Dauno). C4 consisted of 36 accessions (n=5 LR, n=24 OC and n=7 MC) and related, in addition to C2, also to C5. The seven modern cultivars grouped in C4 were all Italian genotypes: Antas, Castello, Ciccio, Ciclope, Fortore, Lesina and Platani, characterized by having Capeiti or Capeiti8 in their pedigree. Antas was related with Capeiti8 by Ichnusa (Biancale × Capeiti8) cultivar parent. Strampelli selections as Senatore Cappelli (or Cappelli), Aziziah and Tripolino clustered in this group together with OC that were Cappelli related. Cappelli was selected from the North Africa population “Jeanh Rhetifah” and is still considered one of the most relevant ancestors of the modern durum wheat cultivars.

C5 consisted of 34 out of 41 MC and was related to C4. All the France, Spain and USA MC and OC clustered in C5 together with 20.6% of the Italian durum wheat accessions.

In order to estimate the divergence between the clusters identified by DAPC analysis, pairwise FSTs were calculated (**Figure 1D**). The higher value (FST=0.49) was found between C1 (all Timilia accessions) and C3 (Dauno group), in agreement with DAPC analysis.

**Figure 2:**
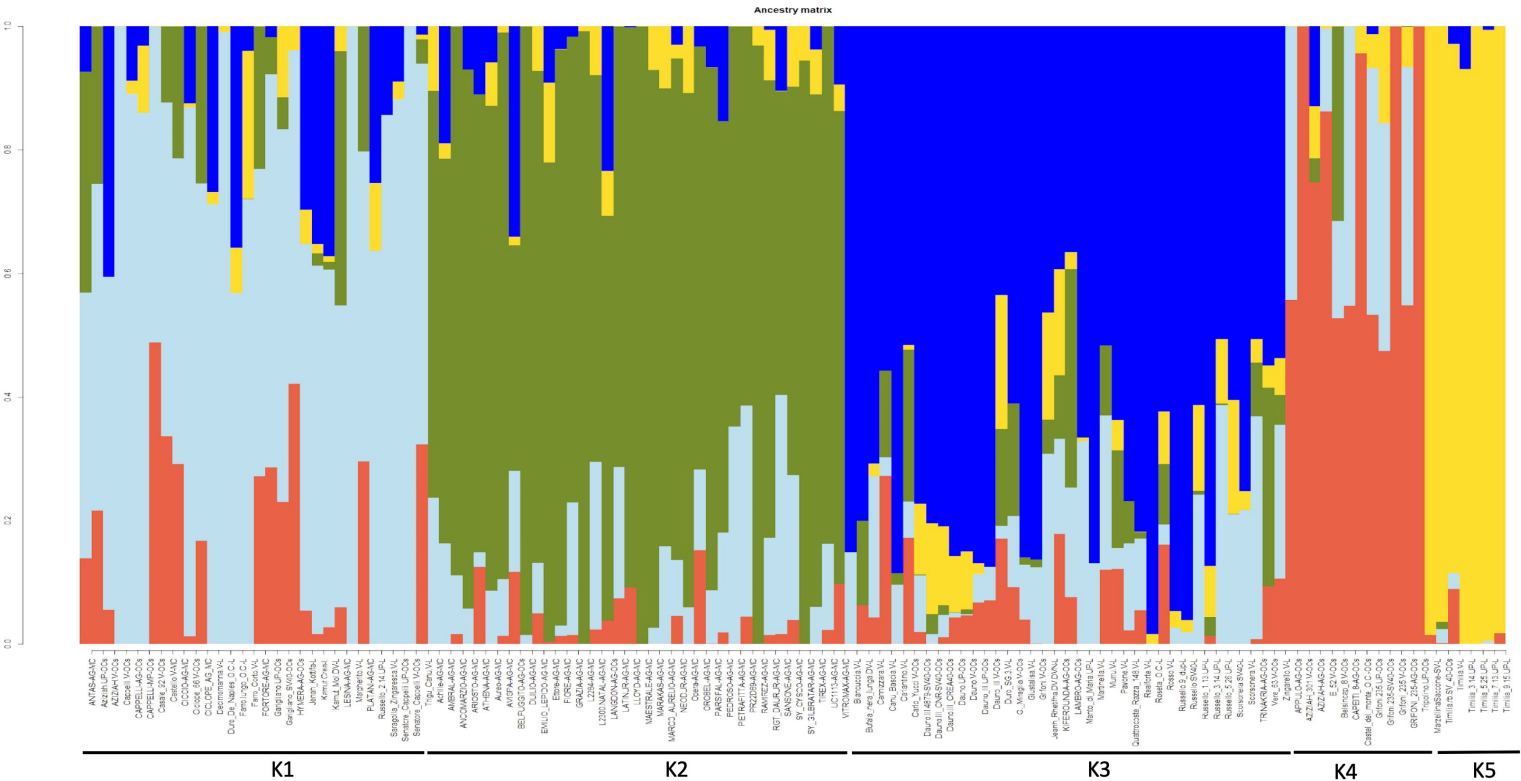
Bayesian assignment analysis as implemented by LEA package (an R package for Landscape and Ecological Association Studies) based on 1907 SNPs data obtained from 123 ex situ durum wheat accessions including 41 landraces, 41 old and 41 modern cultivars.

To better describe the population structure of the durum wheat collection, the *snmf* analysis was performed with the LEA package, **Figure 2**. The barplot provided information on the level of admixture in the collection. At K=5 the barplot differentiated five subpopulations. The first subpopulation (K1) was composed of 30 accessions (11 LR, 12 OC and 7 MC). This group contains all the accessions of Cappelli and its ancestor Jeanh Rhetifah, and only two of these resulted in no admixture accessions: Cappelli_V_OCs and Margherito (LR), both donated to CREA from the Department of Wheat Genetic Resources of the All-Russian Research Institute of Plant Genetic Resources (V labelled=VIR). The second subpopulation (K2) consisted of 36 accessions of which 94% were MC and the remaining part two OC, namely the Italian Belfuggito and the US Langdon. In addition, K2 grouped all the foreign cultivars. The third group (K3) was composed of 38 accessions (25 LR and 13 OC); all the Russello (n=5) and Dauno (n=2 Dauno and n=5 Dauno III) accessions belonged to this group. In both K2 and K3 subpopulations there were no pure accessions. The fourth and fifth groups were smaller than the other ones. In fact, the fourth group (K4) was composed of only 12 OC accessions. In K4 there were the progenitors of a lot of cultivars belonging to this collection such as Grifoni 235 (n=4) and Capeiti 8. In this group Aziziah301, Grifoni235 and Tripolino displayed no admixture structure. Finally, seven accessions were grouped in K5: all Timilia (n=6) and Marzellina Saccone; Timilia 5.25.UP was the only pure accession too and the furthest Timilia accession from DAPC cluster C2.

### 3.2 Morphological characterization of the collection

To identify the relationship between molecular SNP diversity and phenotypic traits of agronomic interest, a field phenotyping trial was conducted in two environments (Foggia and Metaponto). A total of 33 agro-morphological traits were scored: 18 quantitative and 15 qualitative (UPOV-related) traits.

#### 3.2.1 Agro-morphological traits

The estimates of genetic parameters, obtained by mixed ANOVAs, for 18 quantitative agro-morphological traits are presented in **Table 4** and **Supplemental Materials 2-S2**, **S3**. The Phenotypic coefficient of variation (CVp) ranged from 6.43% for kernel width to 147.65% for blackstain-blackpoints, while the Genotypic coefficient of variation (CVg) ranged from 1.03% for grain protein content to 68.80% for blackstain-blackpoints. The phenotypic variance (σ^2^_p_) for all the 18 quantitative traits was always higher than genotypic variance (σ^2^_g_) as well as CVp when compared to CVg, indicating the influence of the environment and requiring multi-environmental selection to maximize genetic values. The CVg and CVp discrepancies were higher for blackstain-blackpoints, yellow berry, number of kernels per spike and weight of kernel for spike, showing their larger environmental plasticity than the other traits. Notwithstanding, the estimates of CVg were closer in magnitude to phenotypic ones for kernel length, plant height, kernel width, total carotenoid content and kernel area.

**Table 4A:** Estimates of variance components and heritability for 18 quantitative agro-morphological traits detected on 123 *ex situ* durum wheat accessions including 41 landraces, 41 old and 41 modern cultivars.

Trait	Genotypic variance	GEI variance	Residual variance	Phenotypic variance	% of Phenotypic variance	h^2^	CV_g_	CV_p_
**Heading date (days from 1st April)**	33.80	9.69	7.30	50.80	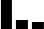	0.85	18.80	22.99
**Plant height (cm)**	502.00	51.90	45.60	600.00	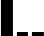	0.94	23.80	26.06
**Awn length (cm)**	4.02	2.59	1.39	8.00	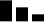	0.73	14.20	20.09
**Spike length cm)**	2.46	0.29	1.47	4.22	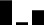	0.86	17.10	22.35
**Spikelets number/spike (n)**	2.31	0.48	3.67	6.46	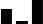	0.73	7.15	11.95
**Number of kernels/spike (n)**	0.78	59.30	78.20	138.00	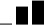	0.02	1.79	23.90
**Weight of kernels/spike (g)**	0.09	0.22	0.33	0.63	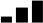	0.36	12.10	31.95
**1000 kernels weight (g)**	19.70	13.40	33.30	66.40	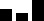	0.62	9.92	18.23
**Kernel roundness**	0.0003	0.00004	0.003	0.003	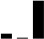	0.43	3.14	9.40

Genotypic, Genotypic x Environment Interaction (GEI), Residual and Phenotypic variance, plot indicating the percentage of the observed component of phenotypic variance, h^2^=heritability in broad-sense (%), CVg=genotypic coefficient of variation (%), CVp=phenotypic coefficient of variation (%).

The broad sense heritability (h^2^) for the studied characters could be divided into four categories as Gharib et al. (2021) suggested: low (less than 40%), intermediate (40-60%), moderately high (60-80%) and very high (more than 80%) heritability. Based on this division, for six traits namely plant height, total carotenoid content, kernel length, spike length, heading date, and kernel area, a very high heritability was observed. The estimates suggested that these traits could be selected directly across seasons with relatively high efficiency. Other six traits (kernel width, number of spikelets for spike, awn length, sedimentation SDS test, yellow berry and 1000 kernels weight) displayed moderately high heritability, instead intermediate and low values were found, respectively, for blackstain-blackpoints, kernel thickness, kernel roundness, weight of kernels for spike, grain protein content and number of kernels for spike as shown in **Tables 4A, B**.

**Table 4B:** Estimates of variance components and heritability for 18 quantitative agro-morphological traits detected on 123 *ex situ* durum wheat accessions including 41 landraces, 41 old and 41 modern cultivars.

Trait	Genotypic variance	GEI variance	Residual variance	Phenotypic variance	% of Phenotypic variance	h^2^	CV_g_	CV_p_
**Kernel area (mm^2^)**	1.96	0.44	1.20	3.60	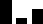	0.82	8.95	12.13
**Kernel length (mm)**	0.21	0.03	0.09	0.33	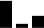	0.88	6.37	8.00
**Kernel width (mm)**	0.02	0.00	0.02	0.04	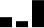	0.74	4.00	6.43
**Kernel thickness (mm)**	0.009	0.003	0.03	0.05	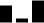	0.55	3.33	7.56
**Blackstain Blackpoints (%)**	0.001	0.001	0.003	0.005	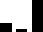	0.57	68.80	147.65
**Yellow berry (%)**	0.15	0.09	0.26	0.50	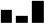	0.63	44.60	81.11
**Grain Protein content (%)**	0.02	0.87	1.57	2.46	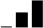	0.03	1.03	10.66
**Sedimentation SDS test**	0.07	0.03	0.11	0.21	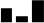	0.68	11.10	19.27
**Total Carotenoid Content (%)**	1.34	0.10	0.46	1.90	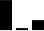	0.91	16.30	19.39

Genotypic, Genotypic x Environment Interaction (GEI), Residual and Phenotypic variance, plot indicating the percentage of the observed component of phenotypic variance, h^2^=heritability in broad-sense (%), CVg=genotypic coefficient of variation (%), CVp=phenotypic coefficient of variation (%).

The differences among the means of the 18 quantitative agro-morphological characters were tested by a nested analysis of variance (ANOVA) based on different sources of variation: between environments (Foggia and Metaponto), between DAPC clusters among the environments, between accessions or genotypes among the clusters and environments, **Table 5**.

**Table 5:** Analysis of variance (ANOVA) of 123 ex situ durum wheat accessions including 41 landraces, 41 old and 41 modern cultivars based on 18 quantitative agro-morphological traits comparing the two environments, the five clusters identified by DAPC, and the genotypes within clusters and environments.

Traits	Code	Between Environments	Between DAPC Clusters (Environments)	Between Genotypes (DAPC Clusters X Environments)
Heading date (from 1st April)	HD	1235.48 ***	1479.82 ***	61.72 ***
Plant height (cm)	PH	9727.40 ***	27711.41 ***	550.20 ***
Awn length (cm)	AwnLen	83.90 ***	107.77 ***	14.95 ***
Spike length (cm)	SpkLen	33.05 ***	72.68 ***	6.11 ***
Spikelets number/spike (*n*)	SpktSPK	91.55 ***	69.01 ***	8.59 ***
Number of kernels/spike (*n*)	KerSPK	213.94 ns	679.47 ***	213.73 ***
Weight of kernels/spike (g)	KerWgtSPK	55.90 ***	5.64 ***	0.96 ***
1000 kernels weight (g)	TKW	9635.49 ***	874.89 ***	89.88 ***
Kernel roundness	KerRou	0.04 ***	0.002 ns	0.003 ns
Kernel area (mm^2^)	KerAre	65.92 ***	62.03 ***	5.33 ***
Kernel length (mm)	KerLen	4.28 ***	3.32 ***	0.59 ***
Kernel width (mm)	KerWid	4.17 ***	0.83 ***	0.05 ***
Kernel thickness (mm)	KerTck	2.82 ***	0.47 ***	0.05 ***
Blackstain blackpoints (%)	BP	0.007 ns	0.02 ***	0.008 ***
Yellow berry (%)	YBer	18.70 ***	4.04 ***	0.76 ***
Grain Protein Content (%)	GPC	80.20 ***	10.48 ***	3.58 ***
Sedimentation SDS test	SDS	16.20 ***	3.16 ***	0.25 ***
Total Carotenoid Content (%)	TCC	127.39 ***	39.48 ***	2.84 ***

(*** for p<0.001. ns= not significant).

Differences between environments were highly significant (p<0.001) in 16 out of 18 traits except for number of kernels per spike and blackstain-blackpoints. Kernel roundness was not significant for the other sources of variation.

**Figure 3:**
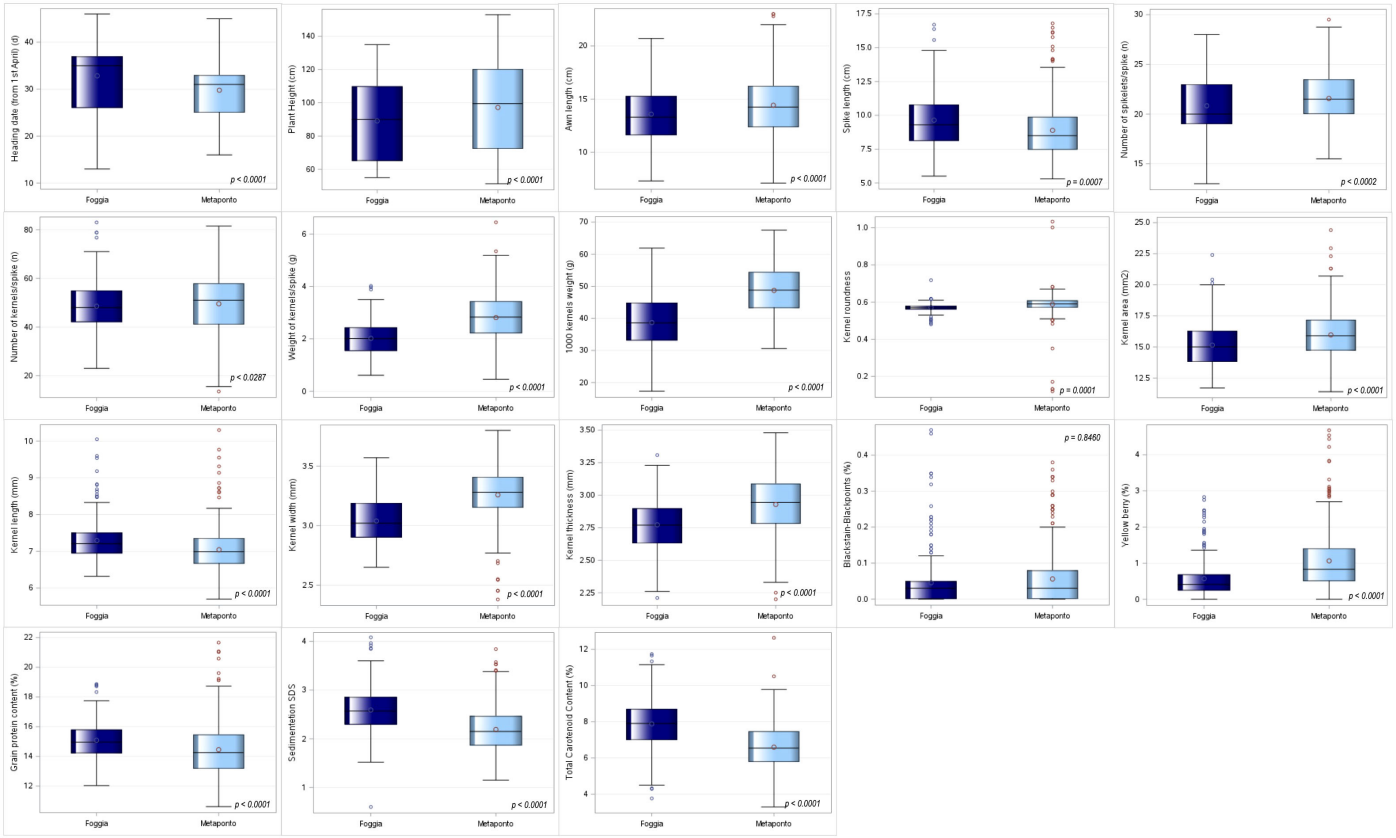
Box-plot distribution based on 18 agro-morphological traits detected on the ex situ durum wheat collection and compared by environments (Foggia and Metaponto).

Since the analysis of the ANOVA showed significant differences between environments, it was decided to proceed by analyzing the two environments separately. The means for each of the 18 quantitative agro-morphological traits in both the environments were computed and then compared (**Supplementary Materials 2**-**S4**, **Figure 3**). Twelve mean values resulted significantly higher in Metaponto than in Foggia, while the latter environment displayed the highest means for heading date, spike length, kernel length, grain protein content, sedimentation SDS and total carotenoid content.

**Figure 4:**
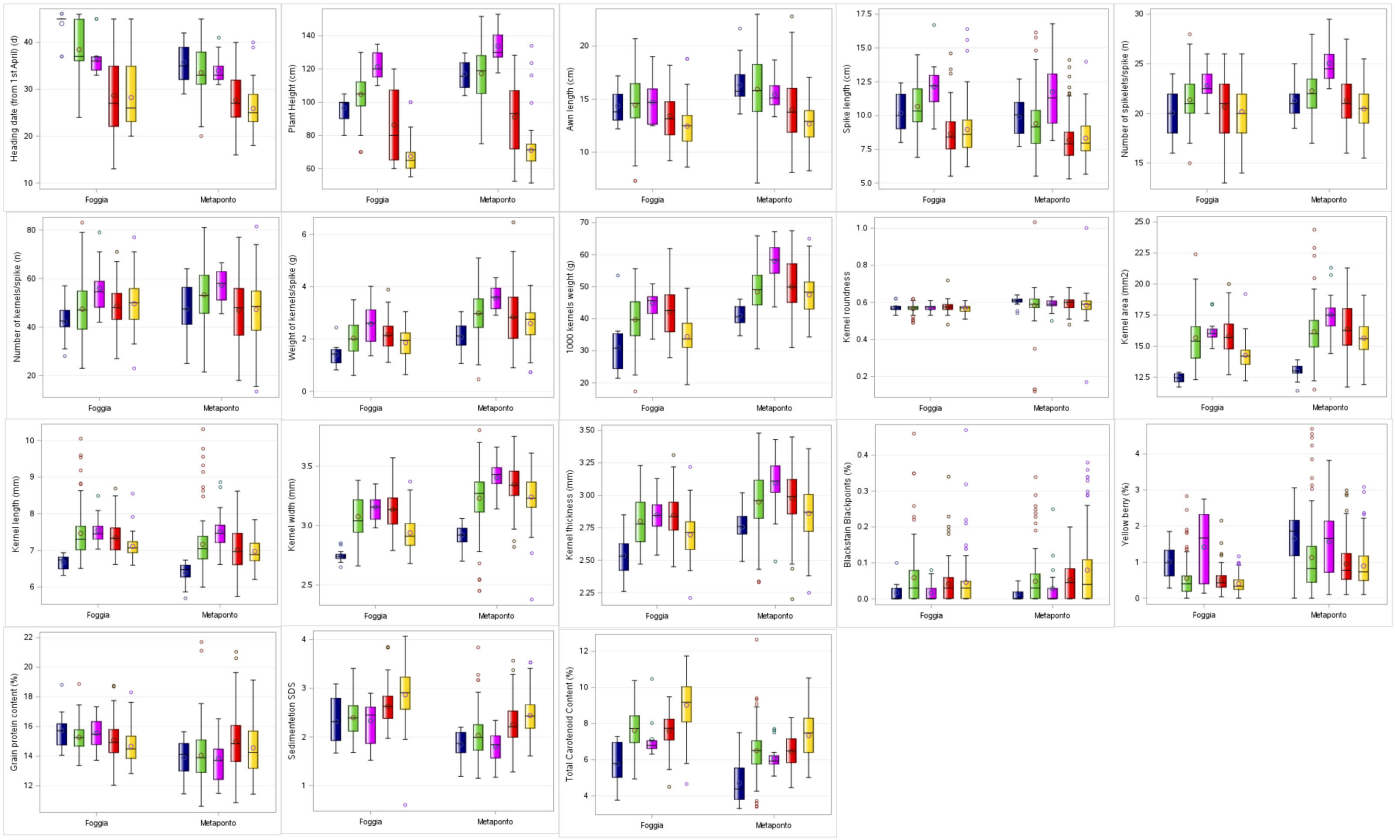
Box-plot distribution based on 18 agro-morphological traits detected on the ex situ durum wheat collection and compared by DAPC clusters in both the environments (Foggia and Metaponto). In dark blue C1, green C2, magenta C3, red C4, gold C5 DAPC cluster.

In addition, descriptive statistics between the group averages obtained by SNP DAPC analysis were reported (**Supplementary Materials 2**-**S5**; **Figure 4**). The C3 DAPC cluster (in magenta) showed the highest mean for 50% of traits in both environments, in contrast to the C1 DAPC cluster (in dark blue). It was noted that the C5 group (in gold) collected the lowest mean values for heading date, plant height and awn length.

#### 3.2.2 UPOV descriptors

**Figure 5:**
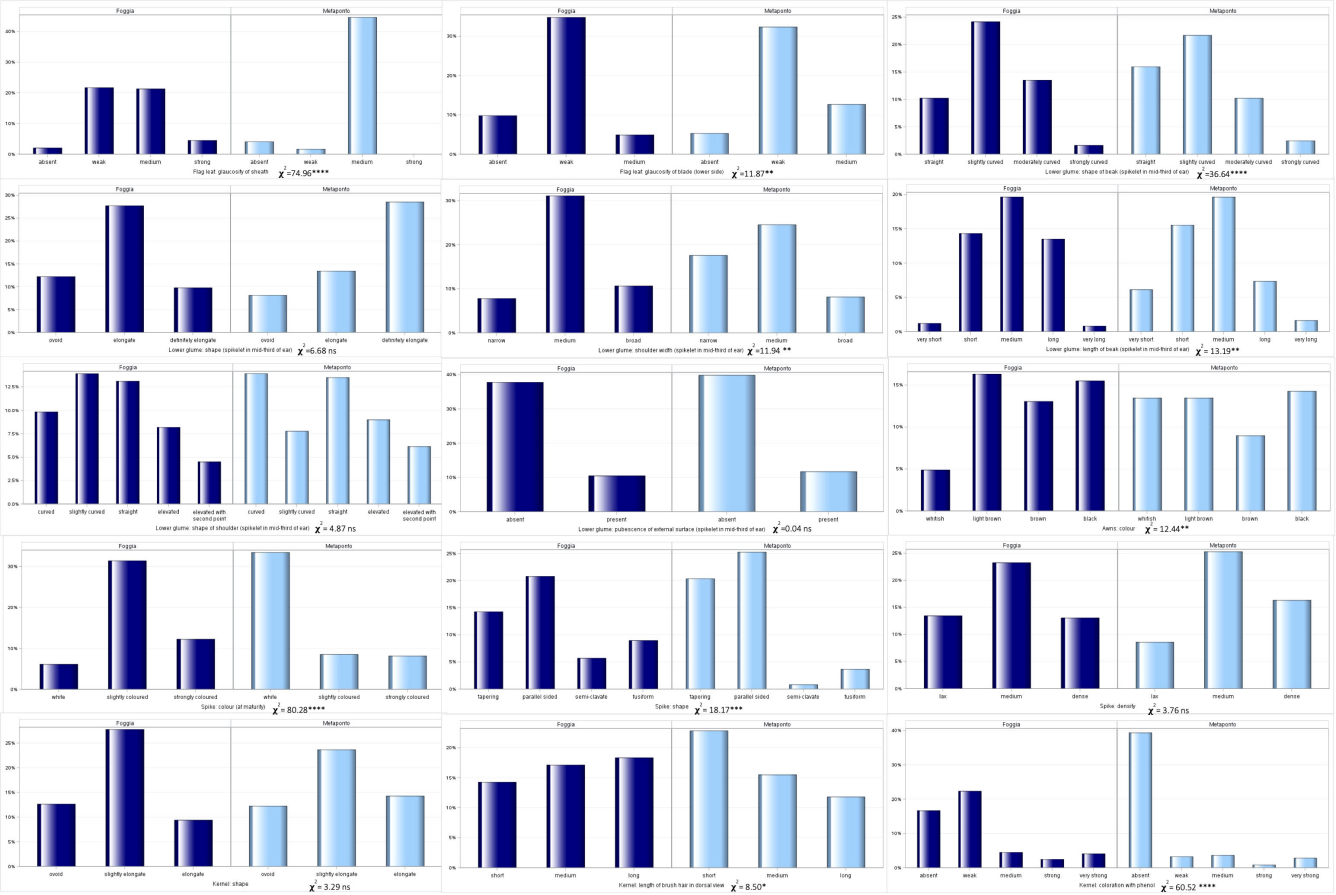
Distribution of the 15 UPOV traits frequencies in both the environments (Foggia and Metaponto).

The 15 morphological traits detected according to descriptors for durum wheat defined by UPOV consisted of ten quantitative (QN), four pseudo-qualitative (PQ) and one qualitative (QL) parameters, expressed in discontinuous states and not influenced by the environment. Their frequencies and differences were analysed (**Figure 5**; **Table 6**) in both the environments.

**Table 6:** Normalized Shannon’s diversity index (H’) estimates for UPOV traits frequencies in the collection of 123 *ex situ* durum wheat accessions across the two environments.

Environment	Foggia	Metaponto
UPOV trait		
Flag leaf: glaucosity of sheath	0.77	0.38
Flag leaf: glaucosity of blade (lower side)	0.72	0.79
Lower glume: shape (spikelet in mid-third of ear)	0.90	0.88
Lower glume: shape of shoulder (spikelet in mid-third of ear)	0.95	0.97
Lower glume: shoulder width (spikelet in mid-third of ear)	0.83	0.92
Lower glume: length of beak (spikelet in mid-third of ear)	0.77	0.86
Lower glume: shape of beak (spikelet in mid-third of ear)	0.82	0.86
Lower glume: pubescence of external surface (spikelet in mid-third of ear)	0.79	0.77
Awns: color	0.94	0.99
Spike: color (at maturity)	0.92	0.79
Spike: shape	0.92	0.70
Spike: density	0.96	0.92
Kernel: shape	0.89	0.96
Kernel: length of brush hair in dorsal view	0.99	0.97
Kernel: coloration with phenol	0.80	0.49
*mean ± SE*	0.86 ± 0.02	0.82 ± 0.05

Test based on UPOV trait frequencies between the two environments. Diversity values: high (H’≥0.6); medium (0.4<H’<0.6); low (H’≤0.4), (Eticha et al., 2005).

All levels of expression for each UPOV descriptor were observed in the whole durum wheat collection, except for the glaucosity of sheath and the glaucosity of blade (lower side) of flag leaf, and the shape of the spike. Ten traits showed significant diversity for their frequencies in the two environments (chi-square test, **Figure 5**). As expected, the pubescence of the external surface (spikelet in mid-third of ear) of lower glume, the QL UPOV descriptor, was not significantly different. The glaucosity of sheath of flag leaf, the color of the spike and the kernels coloration with phenol resulted different in a very highly significant manner (p<0.0001) when considering UPOV QN traits, as well as the shape of lower glume and the color of the spike among UPOV PQ parameters. The shape of beak of lower glume, the density of the spike and the shape of the kernel, although QN variables, were not significantly diverse. Furthermore, each UPOV descriptor showed the same number of classes in the two environments except for the glaucosity of sheath of flag leaf (χ2=74.96; p<0.0001) detected in Metaponto, where the strong class was not observed.

The normalized Shannon’s diversity index (H’) was then determined for all UPOV descriptors in both the environments. Overall, high levels of polymorphism were displayed in each environment, except for the glaucosity of sheath of flag leaf, and the kernels coloration with phenol (H’ = 0.38 and 0.49 respectively) in Metaponto, as shown in **Table 6**. The diversity of Foggia estimates ranged from 0.72 for the glaucosity of blade (lower side) of flag leaf, to 0.99 for the length of brush hair in dorsal view of kernel; in Metaponto, the range was from 0.38 for the glaucosity of sheath of flag leaf to 0.99 for the color of the awns.

**Figure 6:**
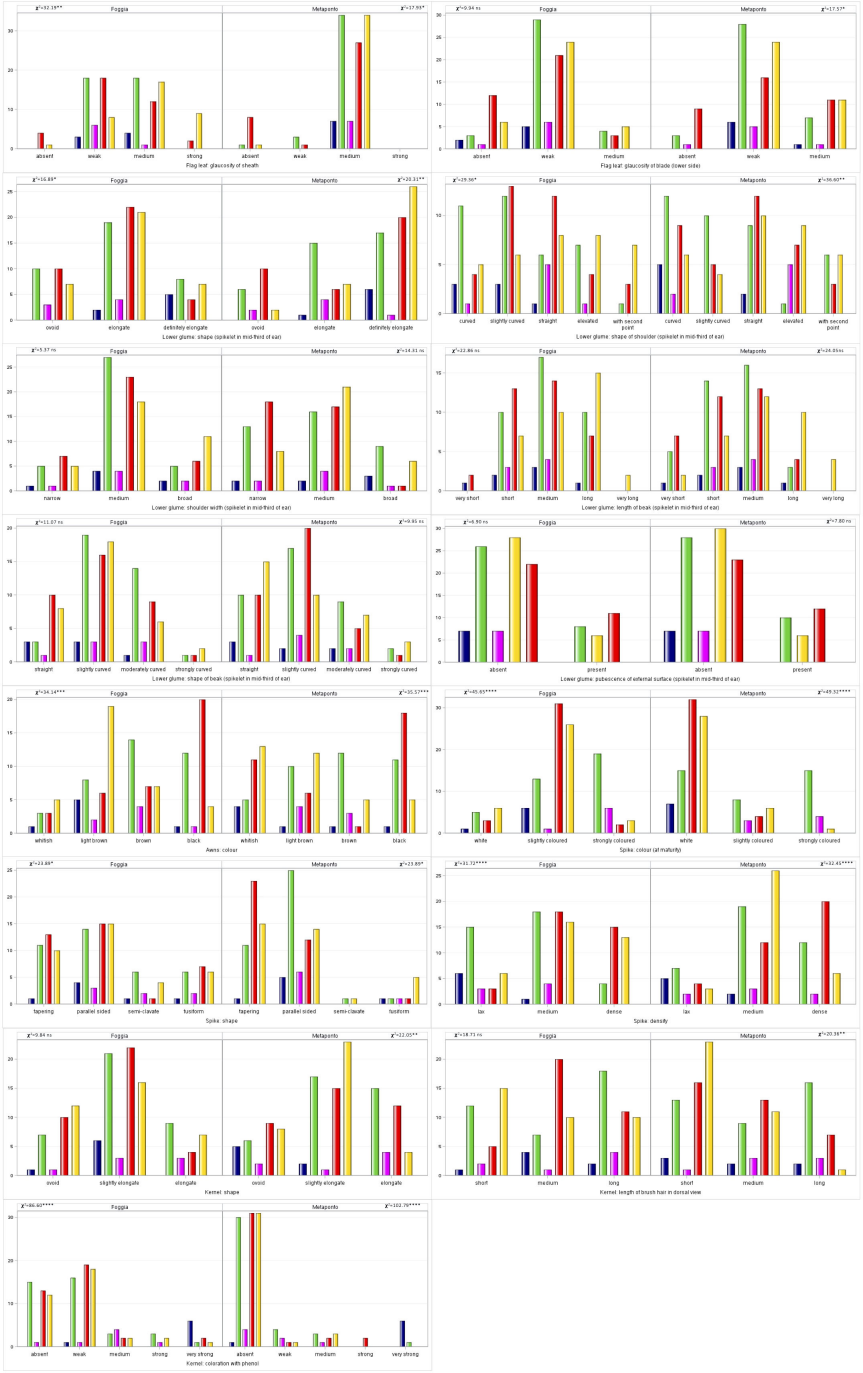
Distribution of the 15 UPOV traits frequencies by DAPC clusters in both the environments (Foggia and Metaponto). In dark blue C1, green C2, magenta C3, red C4, gold C5 DAPC cluster.

With regard to the frequencies of the 15 UPOV descriptors compared between the SNP DAPC clusters, the traits of the awns and the spike, such as color and density, and the kernels coloration with phenol were highly significant diverse (p<0.0001), **Figure 6**.

#### 3.2.3 Morphological traits distinctiveness

To analyze the morphological traits distinctiveness for all the 33 morphological traits, a PCAmix multivariate analysis was carried out.

**Figure 7:**
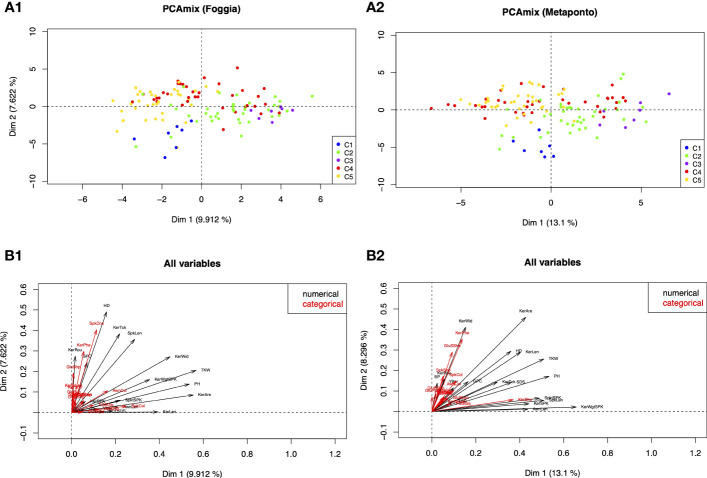
Principal components analysis of mixed data (PCAmix). **(A1, 2)** PCAmix of the first two eigenvectors based on 33 morphological and UPOV traits detected on 123 ex situ durum wheat accessions, grown in two environments (Foggia and Metaponto) and color-coded by DAPC SNPs Clusters. **(B1, 2)** Plot of the squared loadings of all variables (black=quantitative variables; red=qualitative variables).

The results of the PCAmix revealed that the durum wheat collection grown in the two environments of Foggia and Metaponto expressed a similar phenotype. In detail, the distribution of the accessions (**Figures 7A, B**) was explained by the correlation circle that showed the correlation according to the quantitative traits and the levels according to qualitative ones (**Figure 7, A1, A2, B1, B2**, **Supplementary Materials 2**-**S6**, **7**). Among the quantitative traits, plant height, heading time, spike length, weight of kernels per spike and the seed related traits measured (area, length and width) had heavy weight on the differentiation of the groups in both environments. Among the UPOV descriptors, only the kernels coloration with phenol contributed moderately in the two environments while the remaining traits discriminated the accessions to a little extent.

The analysis also revealed that in Foggia the first two dimensions explained the 17.53% of total variance: the first dimension accounted for 9.91% (**Figure 7, A1**) and the second one accounted for 7.62%. In Metaponto the total variance explained by the first two dimensions was larger than in Foggia (21.40%) (**Figure 7, A2**); the first dimension explained 13.10% of total variance and the second dimension accounted for 8.30%.

About 58% of the studied accessions were phenotypically stable in the two environments (**Supplementary Materials 2**-**S6**, **S7**). The second quadrant of the PCAmix scatterplot contained the largest number of accessions (n=45 in Foggia and n=43 in Metaponto), mostly MC, while LR were absent or represented only by one accession (Trigu canu, Foggia environment). The LR accessions were located predominantly in the first and fourth quadrants and in the latter, there were no MC. The OC type, on the other hand, was scattered in all four quadrants.

It should be noted that genotypes clustering in C1 (Timilia and Marzellina Saccone accessions) according to the DAPC analysis, showed late heading, medium-short spike length, low 1000 kernels weight and low kernel area; C1 kernel size was low and grains showed very strong coloration with phenol. On the contrary, genotypes grouped in C3 (Dauno and Dauno III, all OC) and most accessions of C2 (such as Russello accessions) were characterized by tall plants, high 1000 kernels weight and high kernel area and size. Most of the accessions in the C5 group, predominantly MC, confirmed the progress of the *Triticum durum* breeding work done over the last decades: medium-short plant height, early heading date and short spike length, with high quality kernel traits (total carotenoid content and sedimentation SDS) (**Supplementary Figures 2**-**S5**).

In the case of C4 group, some accessions as Capeiti8 and its related cultivars shared their phenotype predominantly with C5 accessions; some other C4 genotypes, as Senatore Cappelli and Cappelli, Margherito, Grifoni, Ciclope, with C2 and C3 accessions.

## 4 Discussion

The durum wheat breeding programs over the years were influenced by consumer’s demands and focused on grain quality and productivity (De Vita et al., 2007; Giunta et al., 2007; Taranto et al., 2020). The selection of varieties with superior agronomic performances caused a reduction of genetic diversity and increased the susceptibility to both abiotic and biotic stresses.

Genetic diversity is imperative to provide a robust food security system capable of adapting to recurrent stresses. It is a crucial step in noticing alleles that could be used as a source of novel traits with high yielding, resilience for biotic and/or abiotic stresses, satisfactory productivity or in meeting the end-user demands in plant breeding (Alemu et al., 2020).

Germplasm banks and research institutions keep, multiply and rejuvenate collections of genetic resources of different genepools collected over time in different areas. There is still little information on these materials, passport data are incomplete and still few accessions are included in pre-breeding and breeding programs.

In the present study we compared SNP genotyping and plant phenotyping by agro-morphological traits, UPOV descriptors and kernel-related traits in order to characterize a wheat collection, consisting of three groups (landrace, old and modern cultivar), under two different environmental conditions and into five clusters as suggested by molecular analysis.

All the obtained results will contribute to knowing the amount of variation of the ex situ durum wheat collection, the genetic relationships among groups, the response of the collection to climate changes, and to facilitate both management and use of wheat genetic diversity that has been lost due to selection in the last decades.

### 4.1 Molecular pattern of diversity of the *ex situ* durum wheat collection

The durum wheat collection analysed by DAPC based on SNP markers revealed a clear lowest Bayesian information criterion subdividing the accessions into five groups in accordance with type of material and pedigree data. The distribution of accessions in the clusters was in agreement with Roncallo et al. (2019) and Ganugi et al. (2021). The landraces (mainly clustered in C2) were separated from modern cultivars (mainly clustered in C5), while the old cultivars were dispersed across all clusters. About 83% of modern cultivars belonged to C5 representing a homogeneous genepool (related pedigrees) and about 73% of landraces clustered in C2. In C5 and C2 there were no landraces and modern cultivars, respectively.

Moreover, the landrace accessions in C2 showed genetic differentiation (Fst=0.16-0.19) lower than accessions in C1 and C3. Instead, the OC accessions released from 1914 (Dauno group) to 1973 (Appulo, Belfuggito and Lambro) were part of C4 but were also dispersed in all DAPC groups. Marzario et al. (2018), genotyping by SSR DAPC an ex situ durum wheat collection from Southern Italy, identified six groups and more than 84% of modern varieties (released from 1974 to 2007) clustered together while old and intermediate varieties dispersed into 5 of the 6 groups formed. Ganugi et al. (2021), evaluating the genetic diversity of 265 tetraploid wheat accessions by 21,051 SNP markers, found a strong selection activity into Italian modern varieties gathered in the same cluster and highlighted genetic homogeneity. Our study supports their results obtained with two different classes of markers and highlights the genetic diversity is still conserved in the studied collection (Fst from 0.16 to 0.49) although modern varieties have a narrow genetic base.

Fiore et al. (2022) and Taranto et al. (2022) showed the genetic differentiation of the Timilia group. Accessions fell in a separate cluster distant from the other pool of accessions and far from the old and modern varieties in accordance with the findings of our study. Timilia accessions represent a typical Sicilian wheat of more ancient origin, widespread in the Mediterranean area in the 18th and 19th centuries and keep a distinctive gene pool obtained by a conservative selection (Taranto et al., 2022). The old cultivar Timilia could have been selected from Timilia landraces that in the meantime could be derived from Marzellina Saccone Sicilian landrace as supposed by Mangini et al. (2018). The Dauno group, instead, was a set of durum lines produced by Nazareno Strampelli by crossing unknown parental lines (De Cillis, 1927) more productive than Timilia. They, although clustered with less distance from the landraces, formed a group in their own right. Dauno group was closed to Realforte (Metaponto environment), one of the best ancient Sicilian landraces (De Cillis, 1927), as reported in Motzo et al. (2004), and characterized by high tillering capacity and low lodging susceptibility in spite of its tallness (138 cm as average of the two seasons), and next to Cannizzara landrace, too. The growing of these three landraces, Timilia, Cannizzara and Realforte was already cited from the Agronomist Salvatore Russo Ferruggia in his 1830 essay on “L’agro trapanese e la sua coltivazione” (Curatolo, 2004 - Regione Siciliana, studio agronomico e vegetazionale POR Sicilia 2000-2006).

The structure of the collection, analyzed by *snmf* analysis, at K=5 identified five subpopulations that in part reflected the groups shown by DAPC: both divisions reflected the history of Italian durum wheat breeding with modern cultivars differentiated by old cultivars and landraces.

As Porceddu (1979) showed in his work, a very large number of botanical forms of *T. turgidum* were concentrated in Sicily, while a few botanical forms of *T. durum*, characterized by a very large number of cultivars, originated from the Sirio-Palestinian area and were concentrated in Sardinia. Breeders started to utilize this variation at the end of the last century by selecting and putting into cultivation the best lines from the original landraces, and since the very beginning of the present century by crossing good lines with introduced germplasm.

All accessions of Timilia and Marzellina Saccone were again separate from the other Sicilian landraces such as Russello that clustered all together in K3. K3 also included all the accessions of Dauno, Dauno III and Jeanh Rhetifha, a landrace that even if considered the progenitor of Cappelli, resulted genetically distant from Cappelli and Senatore Cappelli accessions which, indeed, were grouped in K1 in agreement with Marzario et al. (2018); Fiore et al. (2019) and Taranto et al. (2020). The different clustering of the progenitor could be explained by the no attempt to preserve the genetic material in the original form during breeding history.

DAPC and Bayesian analysis based on SNP data confirmed how not all available resources have been utilized to the full for the development of improved varieties. Most probably, the Timilia and Marzellina Saccone accessions were not used due to their low productivity characteristics (small-medium spike length with low number of seeds, low kernel size, etc.), and have been reintroduced only in recent years thanks to some characteristics such as the especially high content of grain protein. Timilia accessions had also a strong coloration with phenol but a low content of carotenoids in the semolina in both the environments. Taranto et al. (2021) demonstrated that the equilibrium between carotenoid biosynthesis and carotenoid degradation, caused by polyphenol oxidases (PPOs), during processing phases influenced browning semolina and pasta color. The coloration with phenol monitored the action of the polyphenol oxidases (PPOs), one of the oxidative enzymes related to carotenoid degradation. Instead, Taranto et al. (2022) showed that this characteristic of Timilia accession involved adaptation mechanisms as peroxidase and lipoxygenase that were particularly suitable in arid Mediterranean areas. Di Francesco et al. (2021) demonstrated that the Timilia accessions showed a high level of expressions of genes involved in response to attacks by fungi, herbivores and pathogens. Thus, Timilia accessions showed different characteristics suitable in marginal areas and to be used in breeding to overcome climate changes.

### 4.2 Morphological pattern of diversity of the ex situ durum wheat collection

Gene banks could be considered complex “libraries” of materials to be properly cataloged with the aim to recover and use them in research and plant breeding (Spagnoletti Zeuli and Qualset, 1993). Our study wishes to make a contribution in that direction. SNP analysis firstly displayed the road map of wheat evolution. The following step was to identify the relationship between SNP and morphological traits of agronomic interest by field phenotyping, in two different environments, through thirty three morphological traits.

The whole durum wheat collection revealed a large amount of variation for morphological (CV from 12% to 36%) and kernel-related traits (CV from 8% to 12% for kernel traits and from 11% to 22% for quality kernel traits) as reported in **Table S1** (**Supplementary Material 2**); furthermore, there were different levels of susceptibility to blackstain blackpoints and yellow berry diseases and UPOV descriptors showed a high level of diversity (**Table 6**).

The broad sense heritability, based on the division provided by Gharib et al. (2021), revealed that the number of kernel per spike (h^2^ = 0.02) and the grain protein content (h^2^ = 0.03) were not a heritability character and highly influenced by the environment (Mariani et al., 1995; De Vita et al., 2007; Taghouti et al., 2010). Low heritability was also the result for the weight of kernels per spike. Conversely, very high values were observed for plant height, total carotenoid content, kernel and spike length, heading date, kernel area. Kernel width, number of spikelets per spike, awn length, sedimentation SDS test, number of spikelets per spike, yellow berry and 1000 kernels weight were characterized by moderate values.

The phenotypic and genotypic coefficients of variability were estimated for each studied trait. It was observed that the estimates of genotypic coefficients of variability were closer to phenotypic ones for all the studied traits but not for weight and number of kernels per spike, blackstain blackpoints, yellow berry and grain protein content. Except for the latter trait, the remaining ones had relatively low environmental effects on their expression. This was important because it means that the phenotypic expression in these traits reflects the genotypic potential and therefore could be helpful for selection purposes. In relation to both genetic and phenotypic variability and heritability, different studies presented similar results (Bassi and Sanchez-Garcia, 2017; Mathew, 2018; Wolde et al., 2019; Gharib et al., 2021; Chegdali et al., 2022). Abdel-Ghani (2008) evaluated nine hundred and twenty lines of durum wheat landraces and seven varieties in two contrasting environments. For the considered morphological traits, they confirmed that, because of high G x L (location) interactions, estimates of CVg and heritability using combined analysis of variance were generally lower in comparison to the variance values computed separately for each of the two test locations (mono-environment). Thereby, they suggested that performing selection under target environments is the best way to improve wheat productivity. Further, Yagdi and Sozen (2009) in order to determine the inheritance of important agronomic and quality traits, studied ten durum wheat advanced lines and one cultivar during three years; in agreement with our results they demonstrated that environmental variance was important for seed number per spike, seed weight per spike, thousand kernel weight, protein content and SDS-sedimentation, while the variance component of genotype x environment was important for protein content and SDS-sedimentation too.

The industries require grains with superior quality traits. As supported by Taghouti et al. (2010), these traits are also influenced by genotype and interaction of genotype and environment (GxE). They tested twelve Moroccan durum wheat cultivars in five locations, representing a range of environments, in three growing seasons. The results indicated significant effects of genotype, environment and GxE for all the quality traits. For the SDS sedimentation volumes SDS sedimentation volumes, the component of variation due to genotype was larger than the one due to the environment, indicating the greater influence of genotypes on these traits. However, for protein content, the effect of the environment was higher than the effect of the genotypes. Thus, for these traits greatly controlled by environmental effects rather than genetics, multiple environmental trials are necessary in order to better understand and determine the protein content of a cultivar or other genetic materials.

Most of the traits evaluated showed significantly higher values in Metaponto than in Foggia. The Foggia environment influenced a better expression of traits such as heading date, spike and kernel length and quality parameters (grain protein content, sedimentation SDS test and total carotenoid content). Significant differences among durum wheat genotypes and their environmental interaction were reported in literature (Singh et al., 2007; Taghouti et al., 2017; Ficco et al., 2020) in agreement with our work.

### 4.3 Kernel related traits

Kernel dimensions and shape are important factors affecting 1000 kernel weight and grain yield in crops (Arriagada et al., 2020; Sun et al., 2020; Suchowilska et al., 2022) and are critical for grain processing and milling. Kernels with large spherical shape are preferred due to their higher milling value. On the contrary, elongated kernels, as in *T. durum*, are not a highly desirable trait in the milling industry. Martín-Gómez et al. (2019) analyzing six wheat taxa (*T. monococcum, T. dicoccum, T. durum, T. polonicum, T. aestivum* ssp. *aestivum* and *T. spelta*) by seed image, observed that the ancestral taxa had more elongated and less round kernels than the modern bread wheat varieties, due to the selection performed in favor of rounded kernels associated with more yield in the milling process. Suchowilska et al. (2022) explored *T. durum* accessions from the National Plant Germplasm System (USA) and compared them with 12 durum wheat cultivars and Kamut® wheat taking into account dimension and shape descriptors. They assumed that the grains would be more elongated in *T. durum* accessions than in the modern intensively-farmed cultivars, but they did not demonstrate that. They evidenced greater differences in color than in shape descriptors between the investigated accessions and cultivars. In our study we considered five kernel size IPGRI descriptors (roundness, area, length, width and thickness) and in addition UPOV kernel shape descriptor. We compared all them between environments and between SNP DAPC clusters within environments. In Foggia environment, the C2 and C4 displayed seeds significantly longer, thicker and with larger area, than C5 (e.g. kernel length, C2, C4 *vs* C5: 7.5, 7.4 *vs* 7.1 mm (**Supplemental Materials 2**-**S5** and **Figure 4**), while in Metaponto the seed traits were found not to be statistically diverse. In this connection other studies reported differences between LR and elite cultivars (Belhadj et al., 2015; Marzario et al., 2018; Takac et al., 2019; Gharib et al., 2021; Ouaja et al., 2021). The variability of kernel traits (CV, **Supplementary Material 2**-**S5**) was higher for C2 when compared to C5 in both environments. This could confirm in part that over the years, genetic selection for the development of modern cultivars could have narrowed the genetic base of durum wheat even with regard to seed-related traits. In addition, C2 also included two accessions of *T. turgidum* ssp. *turanicum* (Kamut CREA and Kamut Mol) that were different from *T. turgidum* ssp. *durum* ones, especially for high length and area of kernel and low kernel roundness. On the contrary, the Timilia accessions (C1) confirmed the lower 1000 kernels weight and lower mean values for all the seed morphological parameters detected in comparison to the other LR (C2) considered in the study, according to the results from De Santis et al. (2017).

### 4.4 Variation based on UPOV descriptors

The UPOV protocols were important to protect and distinguish crop varieties. Generally, no single morphological trait could be used to distinguish cultivar so these protocols studied the appropriate combination of traits useful to better characterize the varieties. In this study 15 UPOV-defined traits were detected to describe the durum wheat collection. Their frequencies underlined the significant differences between environments and DAPC groups within them. Five out 15 UPOV traits were stable between the environments (**Figure 5**), while only nine traits exhibited significant differences between DAPC clusters in both the environments. For length of beak of lower glume, C2 and C4 showed mainly the classes from short to medium while C5 from medium to very long ones, with this last class detected only in C5 in both the environments. Spike density reflected the breeding evolution, in both the environments; it was evident how the landraces were characterized by a prevalence of medium and lax spike density while C4 and C5 had a prevalence of medium and dense spike density. This trait was an important morphological character associated with wheat grain yield. In fact, in the past years the breeders selected wheat varieties with longer and more compact spikes because it was a feasible way to increase the number of kernels per spike and therefore to improve the grain yield (Liu et al., 2020). Glaucosity of sheath and glaucosity of blade of flag leaf were among the UPOV descriptors that showed differences in only one environment. They were proved to be useful traits in distinction and identification of wheat varieties, but the unclear time and detection procedure in the UPOV document TG/1/3 for durum wheat could affect their detection and therefore are not involved in identification breeding programs (Shahaji et al., 2020). The glaucosity was associated with several traits mainly related to an increased drought, heat tolerance and higher yield under dry conditions (Würschum et al., 2020) since it reduces cuticle permeability, water loss, photosynthetic temperature and reflects short-wave radiation (Gharib et al., 2021). However, the plant’s response to a given stress was a complex process and the presence or absence of glaucousness is only one of the components (Würschum et al., 2020). Among the 15 tested descriptors, coloration of kernels with phenol significantly discriminated genotypes between DAPC clusters with the C2 being more heterogeneous than C5. Taranto et al. (2021) demonstrated that the equilibrium between carotenoid biosynthesis and carotenoid degradation, caused by polyphenol oxidases (PPOs), during processing phases influenced browning semolina and pasta color. Our study confirmed that over time the durum wheat breeding selection has developed MC with higher total content of carotenoid than LR and OC. Coloration of kernels with phenol monitored the action of the polyphenol oxidases (PPOs), one of the oxidative enzymes related to carotenoid degradation, and its lower and significant value in MC (Foggia C5, H’=0.60 and Metaponto, H’=0.38) was in accordance with Taranto et al. (2021): to obtain high-quality product acceptable to consumers, the enzymatic browning process must be necessarily controlled. The differences between the environments could be due to the type of expression of the parameter defined by TGP/10/2 (https://www.upov.int/edocs/tgpdocs/en/tgp_10.pdf). All UPOV traits, except for pubescence of external surface of lower glume, qualitative trait, were quantitative or pseudo-qualitative ones and for them the level of variation due to the environment can differ from genotype to genotype, especially in heterogeneous landraces, and from characteristic to characteristic. In conclusion, UPOV protocols provided basic information of the accessions and it would be desirable to use traits able to distinguish the varieties and stable over repeated propagations of such a variety. But in our study the only UPOV traits did not show a clear discrimination among the collection and, also, some traits were difficult to detect so it would be useful to review the parameters to be used for the description in agreement with those reported by Giunta et al. (2007); Pagnotta et al. (2013) and Shahaji et al. (2020).

### 4.5 Comparing the durum wheat genetic resources with pedigreed varieties

Thagouty et al. (2017) studied a set of 29 durum wheat genotypes grown in Morocco, released in different periods during the 20th and the beginning of 21st centuries, in order to quantify the achievement of past and present Moroccan breeding efforts. They showed that the sowing-heading phase decreased in modern cultivars allowing them to escape to higher air temperature and drought stresses accentuated by climate change and confirmed previous reported evidences on Spanish and Italian genotypes as in De Vita et al. (2007); Motzo and Giunta (2007) and Isidro et al. (2011).

The breeders’ work has driven the development of cultivars that are highly stable across diverse environments and nowadays more resilient than the past. De Vita et al. (2010) evaluated 65 durum wheat genotypes, released or grown in Italy, in three locations across Italy, over four growing seasons. They proved that old cultivars were characterized by a minimal responsiveness to improved environmental conditions, showing an almost stable nominal yield in agreement with the concept of “biological” or “static” stability. In contrast, the modern cultivars were highly responsive to fertility improvements and showed a pronounced adaptation to high-input environments. Thus, since the breeding strategies adopted during the last decades contributed to reduce the interaction of genotypes with environments selecting genotypes with better stability across a wide range of locations and years, modern genotypes outperformed the old ones in all test environments with a strong adaptability. In our study the PCAmix analysis based on 33 morphological traits displayed that about 58% of genotypes were stable across environments and in all three germplasm types, also confirming the DAPC and STRUCTURE analyses, where modern cultivars were grouped in clusters C4 and C5 or K1 and K2, respectively. The stable MC phenotypes were validated by the PCAmix analysis, in fact they were predominantly distributed in the second quadrant (24 MC in the II and 1 MC in the I quadrant). Simultaneously, the phenotypic variability of both landraces and old cultivars was confirmed by their dispersion in the plot.

## 5 Conclusion

A huge variability was detected at morphological level, with landraces being more diversified than modern varieties. The data collected in this study provide useful resources to facilitate both management and use of wheat genetic diversity that has been lost due to selection in the last decades.

The analysed ex situ durum wheat collection provides an opportunity for supporting the protocol for official examination of Distinctness, Uniformity and Stability (DUS) for durum wheat varieties entered for National Variety List (NVL) and Plant Breeders’ Rights. Furthermore, the phenotypic and genotypic data acquired for the genetic materials under threat of genetic erosion (i.e. landraces and old varieties) could be used to start the administrative procedure for the registration to the NVL as “conservation variety”.

## Data availability statement

The original contributions presented in the study are included in the article/**Supplementary Material**. Further inquiries can be directed to the corresponding author.

## Author contributions

Conceptualization: TG, GL, SM, SE, FF, FT, and PV. Methodology:SM, GL, TG, and PV. Supply of materials: PV. Analyzed data: SR, SM, and GL. Writing—original draft preparation: SR and GL. Writing—review and editing: SM, GL, TG, and PV. Supervision: TG, GL, and PV. Contributed to the article and approved the submitted version: all authors.
